# 4,5-Dimethoxy-2-nitrobenzohydrazides and 1-(1-Benzylpiperidin-4-yl)ethan-1-ones as Potential Antioxidant/Cholinergic Endowed Small Molecule Leads

**DOI:** 10.3390/scipharm86010002

**Published:** 2017-12-21

**Authors:** Rukhsar Banu, Jason Gerding, Cynthia Franklin, Donald Sikazwe, William Horton, Marianna Török, Julian Davis, Kwan H. Cheng, Muziya Nakazwe, Bereket Mochona

**Affiliations:** 1 Pharmaceutical Sciences Department, Feik School of Pharmacy, University of the Incarnate Word, San Antonio, TX 78209, USA; banu@student.uiwtx.edu (R.B.); gerding@uiwtx.edu (J.G.); cefrankl@uiwtx.edu (C.F.); 2 Department of Chemistry, University of Massachusetts Boston, Boston, MA 02125, USA; william.horton001@umb.edu (W.H.); marianna.torok@umb.edu (M.T.); 3 Chemistry Department, School of Mathematics, Science and Engineering, University of the Incarnate Word, San Antonio, TX 78209, USA; judavis@uiwtx.edu; 4 Department of Physics and Astronomy and Neuroscience Program, Trinity University, San Antonio, TX 78212, USA; kcheng1@trinity.edu; 5 Anatomy and Physiology Department, School of Osteopathic Medicine, University of the Incarnate Word, San Antonio, TX 78209, USA; nakazwe@uiwtx.edu; 6 Department of Chemistry, Florida A&M University, Tallahassee, FL 32307, USA; mochonab@yahoo.com

**Keywords:** poly-active, antioxidant, hydrazones, isonipecotates, cholinesterase, in silico

## Abstract

The objective of this research is to generate leads for developing our ultimate *poly-active* molecules with utility in central nervous system (CNS) diseases. Indeed, poly-active molecules capable of mitigating brain free radical damage while enhancing acetylcholine signaling (via cholinesterase inhibition) are still being sought for combating Alzheimer’s disease (AD). We differentiate “poly-active” agents from “multi-target” ones by defining them as single molecular entities designed to target only specific contributory synergistic pharmacologies in a disease. For instance, in AD, free radicals either initiate or act in synergy with other pharmacologies, leading to disease worsening. For this preliminary report, a total of 14 (i.e., 4,5-dimethoxy-2-nitrobenzohydrazide plus 1-(1-benzylpiperidin-4-yl)ethan-1-one) derivatives were synthesized and screened, in silico and in vitro, for their ability to scavenge free radicals and inhibit acetylcholinesterase (AChE)/butyrylcholinesterase (BuChE) enzymes. Overall, six derivatives (**4a**, **4d**, **4e**, **4f**, **4g**, **9b**) exhibited potent (>30%) antioxidant properties in the oxygen radical absorbance capacity (ORAC) assay. The antioxidant values were either comparable or more potent than the comparator molecules (ascorbic acid, resveratrol, and trolox). Only three compounds (**4d**, **9a**, **9c**) yielded modest AChE/BuChE inhibitions (>10%). Please note that a SciFinder substance data base search confirmed that most of the compounds reported herein are new, except **9a** and **9c** which are also commercially available.

## 1. Introduction

Disease-modifying, anti-Alzheimer’s disease (AD) molecules continue to elude both big and small pharma discovery approaches. Part of the problem is that AD pathology is underpinned by errant synergistic or intertwined pharmacologies. Our inclination is that small molecules capable of modulating disease synergistic or intertwined contributory pharmacologies could more effectively modify multi-factorial diseases like AD and slow its progression. 

Our initial search for potentially poly-active small (Formular Weight (FW) < 500) molecule leads possessing free radical, acetylcholinesterase (AChE) suppressive activities has thus far produced compounds with potent radical scavenging capabilities and modest acetylcholine potentiating properties. The pharmacophores utilized (i.e., 4,5-dimethoxy-2-nitrobenzohydrazide or hydrazones and 1-(1-benzylpiperidin-4-yl)ethan-1-one or isonipecotates) were pursued due to their reported multi-pronged attributes, that is, their abilities to donate or accept hydrogen and exert their own biological effects [1,2,3,4,5]. The fact that they can be readily derivatized with diverse hetero-/non-hetero aromatic groups to afford new chemical entities possessing advantageous pharmacological profiles is a bonus [6,7,8,9].

The above molecules were designed to structurally contain at least two hetero-aryl functionalities spaced by a 2 to 3 carbonylated atom linker and electrostatically mimic donepezil (**Do**, an AChE inhibitor with clinical utility in all phases of AD) [10,11,12]. They were synthesized in a parallel approach, and tested for their radical and AChE inhibitory extents. Antioxidant or radical scavenging capacities were desired in our molecules because excess reactive oxygen species (ROS), directly (e.g., via protein and lipid oxidations) and indirectly (e.g., via apoptotic or ß-amyloid mechanisms), ravage neurons in AD [13,14,15]. Enzyme inhibition tests were conducted, both in silico (predictively for AChE only) and in vitro (to confirm for AChE/butyrylcholinesterase (BuChE)). Interestingly, cholinesterases continue to be drug design targets in this arena, even though their sole role in AD remains somewhat unclear. For instance, while their inhibition improves the acetylcholine (ACh) signaling for memory/cognition, the two enzymes also contribute to plaque assembly in AD [12,16].

## 2. Materials and Methods

### 2.1. Synthesis

**Scheme 1:**
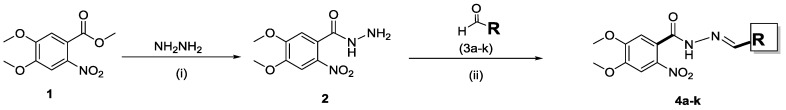
Reagents and conditions: (i) NH_2_NH_2_, EtOH, 60 °C, 60–70%; (ii) Hydrazide, 2,3-dihydrobenzofuran-6-carbaldehyde, EtOH, HCl, room temperature, 70%—quantitative.

**Scheme 2:**
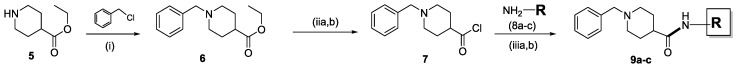
Reactions and conditions: (i) Benzyl chloride, Et_3_N, toluene, room temperature, > 90%; (iia) NaOH, H_2_O, CH_3_OH, reflux, not isolated; (iib) CH_2_Cl_2_, SOCl_2_, reflux, not isolated; (iiia) Et_3_N, NH_2_-Ar, dioxane or CH_2_Cl_2_, reflux, 60–75%; (iiib) HCl-ether, >80%.

**Figure 1:**
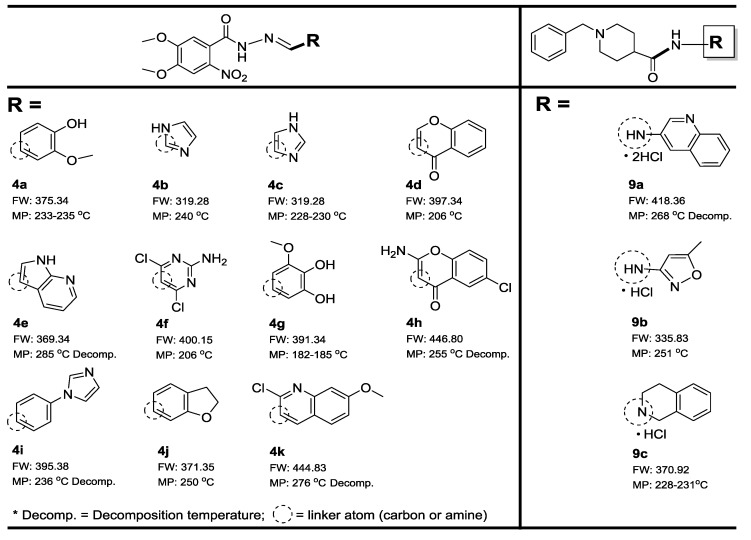
Structures of the synthesized hydrazones (**4a**–**k**) and isonipecotates (**9a**–**c**). Note that FW means Formula Weight and MP denotes Melting Point.

Hydrazone synthesis occurred in two steps (Scheme 1) [6,7,8]. Step one involved refluxing methyl 4,5-dimethoxy-2-nitrobenzoate **1** and excess hydrazine monohydrate in absolute ethanol, and afforded intermediate **2** in yields of 60–70%. In step two, intermediate **2** was condensed with a variety of aromatic aldehydes **3a**–**k** to form arylated hydrazones **4a**–**k** as solids (Figure 1, 70%-quantitative yields). Exploratory arylated isonipecotates **9a**–**c** were prepared beginning with the trimethylamine-facilitated *N*-benzylation of isonipecotate ethyl ester **5** in toluene (Scheme 2) [9]. Reaction yields of the benzylated intermediate **6** were >90%. Ester hydrolysis, under basic reflux, led to the carboxylic acid intermediate, which was promptly converted to the acyl chloride **7** via the dropwise addition of SOCl_2_. Finally, acyl chloride treatment with appropriate amines **8a**–**c**, in step three (Scheme 2), afforded target products **9a**–**c** (60–75% overall yields).

### 2.2. Oxygen Radical Absorbance Capacity (ORAC) Assay

We needed to screen our compounds for their direct radical scavenging capabilities in both lipid and aqueous environments. To that end, we utilized the oxygen radical absorbance capacity (**ORAC**) assay, which measures peroxyl radical scavenging via hydrogen atom transfer (HAT) or electron transfer (ET) to the existing or pre-formed radical. Assays details are well reported [17,18]. Briefly, we utilized known conditions for our assay and 2,2′-azobis(2-amidino-propane) dihydrochloride (AAPH) as the oxidant or peroxyl radical ROS generator [19,20,21]. In our hands, 4.19 µM fluorescein stock solution was prepared in 75 mM phosphate buffer (pH = 7.4, kept at 4 °C), diluted with the same buffer to a concentration of 0.0816 µM, and incubated at 37 °C for 15 min before assaying. A fresh 153 mM AAPH solution in the said buffer was prepared, kept on ice, and used for 4 h at the most. Then, 10 mM solutions of trolox in ethanol, 10 mM solutions of ascorbic acid in water, and 50 mM stock solutions of test compounds in DMSO were prepared, and each was diluted to 80 µM with ethanol. Subsequently, 25 µL of each diluted stock solution or 25 µL of ethanol with 0.16% DMSO in case of control (no test or reference compound) was plated with 150 µL of fluorescein solution, and 25 µL of the above AAPH solution was added to all wells except those for maximum fluorescence control. Thus, the test compounds (**4a**–**k**, **9a**–**c**, and donepezil or **Do**) and the reference compounds (ascorbic acid, resveratrol, and trolox) were all tested at final concentrations of 10 µM in the assay.

**Figure 2:**
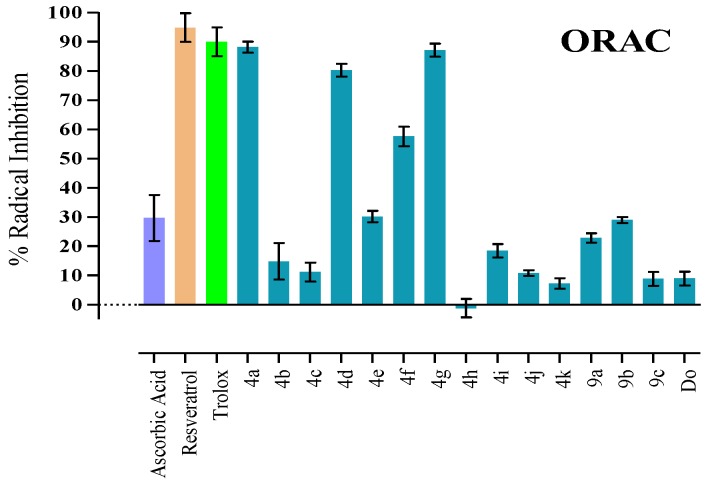
The percentage (%) of radical scavenging of three reference antioxidants (ascorbic acid, resveratrol, and trolox), hydrazones (**4a**–**k**), isonipecotates (**9a**–**c**), and donepezil (**Do**) determined in the oxygen radical absorbance capacity (ORAC) assay after 60 min. Data are expressed as means of the % radical scavenging ± standard deviation (SD), where the number of independent repeats is n = 3 [22].

The maximal fluorescence intensity was obtained by a SpectraMax i3x microplate reader equipped with SoftMax Pro 6.5.1 (Molecular Devices, Sunnyvale, CA, USA) software at an emission wavelength of 520 nm with a preset excitation wavelength of 485 nm. Measurements were taken kinetically every 2 min for 60 min at a constant temperature of 37 °C. Plates were shaken for 5 s before each reading. Measurements were run on multiple plates in triplicate sets. Plates were sealed with a transparent cover to prevent evaporation. Background of the AAPH solution with appropriate amount of DMSO, ethanol, and buffer, but no fluorescein was taken in every plate and used as a blank for all the wells tested. Percent radical scavenging activity was calculated using the expression: **[(*AUC_t_* − *AUC_c_*)/*AUC_f_max_*] × 100%**, where ***AUC_t_*** is the net area under the fluorescence curve obtained in the presence of the test/reference compounds, ***AUC****_c_* is the net area under the fluorescence curve obtained for the control sample that contained no antioxidant (no test/reference compound), and ***AUC_f___max_*** is the net area under the fluorescence curve obtained for the maximum fluorescence control sample that contained no radical and thus had the maximum amount of fluorescein dye. The net area (*AUC*) under the fluorescence curves was determined using the following equation:

$$Net\;AUC=0.5+\underset{0-29}{\sum }\frac{f_{i}}{f_{0}}+\left(0.5*\frac{f_{30}}{f_{0}}\right)$$

where *f*_0_ is the measured fluorescence intensity at time 0 and *f_i_* is the measured fluorescence intensity at time *i*. The ORAC assay percent radical scavenging activities are reported in Figure 2.

### 2.3. In Silico AChE Inhibition

Since the said compounds were designed to mimic **Do**’s structural/electronic and therefore pharmacologic behaviors, molecular dockings of all fourteen ligands were conducted against AChE only. The crystal structure of AChE was derived from the Protein Data Bank database (PDB ID: 1EVE) [11]. Before docking, water molecules and the embedded **Do** ligand were removed from the AChE protein structure. Non-polar hydrogens were added to the protein using AutoDock Tools (Version 1.5.6) software and the correct protonation state of each ligand was determined at pH 7.4 using MarvinSketch (Version 17.2.27 ChemAxon, Cambridge, MA, USA) [23]. To create the optimized 3D structures, we used obconformer—a molecular mechanics modeling program based on the force field MMFF94 from Open Babel [24]. Docking of the ligands to AChE was performed using AutoDock Vina (Version 1.1.2) [25]. Flexible ligand conformations were used in all dockings. For search space, a rectangular box of size 28.5 × 18.75 × 18.75 Å^3^ with its geometrical center set to that of the originally embedded **Do** was used. 

**Figure 3:**
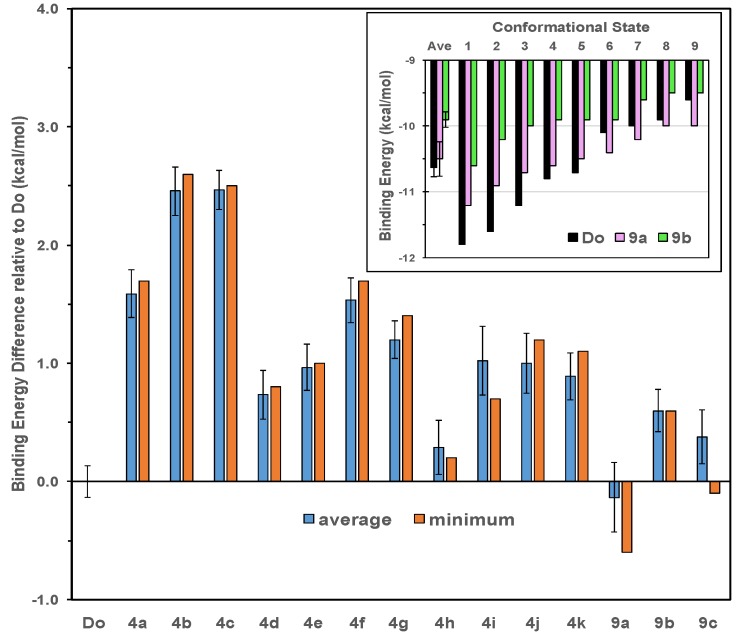
Summary of binding energies obtained for all compounds reported herein including comparative conformations of **Do** versus compounds **9a** and **9b**.

**Figure 4:**
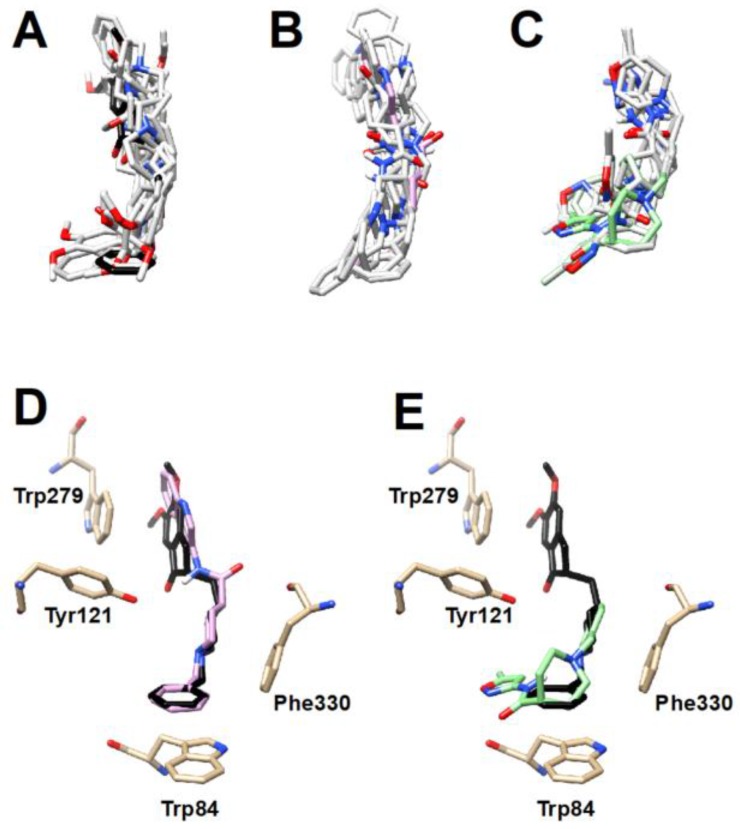
Computational binding of compounds at acetylcholinesterase (AChE). (**A**–**C**) represent the nine predicted conformations while (**D**,**E**) refer to the lowest energy binding orientations obtained by docking.

Nine different conformations (1 to 9) with the binding energies sorted from the lowest to highest binding energy were obtained from the molecular docking, and the energies of the representative ligands, **Do**, **9a**, and **9b**, are shown in Figure 3. Also, the average and the standard error of each ligand are demonstrated for the representative ligands. The average and the minimum binding energies of all fourteen compounds were evaluated, and their values are represented as the binding energy differences, i.e., the binding energy of each ligand minus the binding energy of **Do**; for both, average and minimum energies are also indicated in Figure 3. It is clear that **9a** exhibited the lowest difference in binding energy versus other compounds. Figure 4A–C overlaid structures show the predicted conformations of representative ligands **Do**, **9a**, and **9b**, respectively. Essentially, nine structures of each ligand are superimposed and the ones with the lowest binding energies are highlighted in black, pink, and green, accordingly. Figure 4D and Figure 4E, respectively illustrate the lowest energy structures of **9a** and **9b** versus **Do** in **AChE** active site/gorge. Notably, the lowest energy structure of our docked **Do** closely matched that of the reported crystal structure of embedded **Do** [11]. Specifically, we observed close proximities of the following protein residues with various groups of **Do**: (1) Trp279 to the indanone ring of **Do** via π-π interactions at the proposed entrance to the gorge of AChE; (2) PhE330 and Tyr121 to the nitrogen of the piperidine ring of **Do** via cation-π and hydrogen bonding, respectively, in the middle of the gorge; and (3) Trp84 to the benzyl ring via π-π stacking at the bottom of the gorge, as proposed previously [11].

### 2.4. In Vitro AChE/BuChE Inhibition

To determine compound selectivity, both AChE and BuChE inhibitory studies were undertaken. The two assays were conducted using modified Ellmann’s procedures [26,27,28,29]. Electric eel AChE (catalog number: C2888-500UN) and equine serum BuChE (catalog number: C4290-1KU) were purchased from (Sigma-Aldrich, St. Louis, MO, USA). Enzyme aliquots of 6 U/mL were prepared in 20 mM HEPES buffer (Ph = 8.0) containing 0.1% TritonX-100, stored at −20 °C until use, when they were thawed and diluted 20X with 100 mM phosphate buffer (pH = 8.0). Subsequently, 10 mM Stock solutions of inhibitors (test compounds, galantamine (**Ga**) and **Do**) were prepared in DMSO and then diluted to 0.15 mM through a co-solvent method by adding 145.5 µL of 0.1 M phosphate buffer (pH = 8.0) and 150 µL of acetonitrile to 4.5 µL of 10 mM inhibitor stock solution. 5,5′-dithio-bis-(2-nitrobenzoic acid) or DTNB, also called Ellman’s reagent, stock solution of 0.4341 mM in 100 mM phosphate (pH = 8.0 buffer) was also prepared. Finally, depending on the assay, acetylthiocholine or butyrylthiocholine stock solutions (4.124 mM in 100 mM phosphate buffer, pH = 8.0) were made. These stock solutions were used in the ensuing enzymatic reactions in 96 wells. Ultimately, each well comprised a final assay volume of 150 µL and the following ingredients: 0.34 mM DTNB, 0.02 unit/mL AChE or BuChE, 0.55 mM acetylthiocholine or butyrylthiocholine, and 2 µM inhibitor (except in the case of the control, which had no inhibitor) for AChE or 10 µM inhibitor (except in the case of the control, which contained no inhibitor) for BuChE. Assays were also carried out with a blank solution containing all components except the enzyme and inhibitor so as to account for non-enzymatic reactions. 

**Figure 5:**
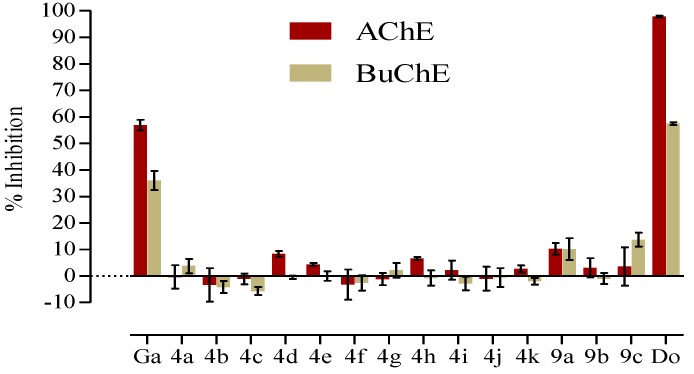
Respective enzyme inhibitions upon exposure to test and reference compounds (galantamine or **Ga** and **Do**). All compounds were tested at 2 µM (AChE) using 0.02 U/mL enzyme quantities. Data are expressed as means of the % AChE inhibition ± SD, n = 3. BuChE: butyrylcholinesterase.

Measurements were run on multiple plates, in triplicates. The substrates acetylthiocholine and butyrylthiocholine were cleaved by their respective enzymes, generating thiol groups detected via their reaction with the colorimetric Ellman’s reagent, DTNB. Initial rate measurements were performed at 37 °C using a VersaMax microplate reader with SoftMax Pro 5 software (Molecular Devices, Sunnyvale, CA, USA) and collecting absorbances at 412 nm every 15 s for 15 min. Percent inhibitions of the enzyme activity due to the presence of test compounds with respect to the control were calculated by the following expression: **[(*v*_0_ − *v_i_*)/*v*_0_] × 100**, where ***v_i_*** and ***v*_0_** are the rates calculated in the presence and absence of an inhibitor. AChE and BuChE act independently to hydrolyze/deactivate acetylcholine, and their inhibition leads to enhancements in the levels and activity of ACh. Enzyme inhibition data obtained from test and reference molecules are displayed in Figure 5.

## 3. Results and Discussion

Overall, 11 4,5-dimethoxy-2-nitrobenzohydrazide or hydrazone plus three 1-(1-benzylpiperidin-4-yl)ethan-1-one or isonipecotate derivatized small molecules (Figure 1, FWs <500) were synthesized and preliminarily evaluated at 10 μM for free radical scavenging abilities, and at 2 μM for cholinesterase inhibition, using established techniques. Compounds **4a**, **4d**–**g**, and **9b** potently scavenged radicals (>30%) in the ORAC assay; that is, they performed at or better than ascorbic acid. In fact, **4a**, **4d**, and **4g** performed comparable to reference compounds trolox and resveratrol. The ORAC differentiation in antioxidant capability is significant because this assay employs radicals with practical relevance in living organisms.

**Figure 6:**
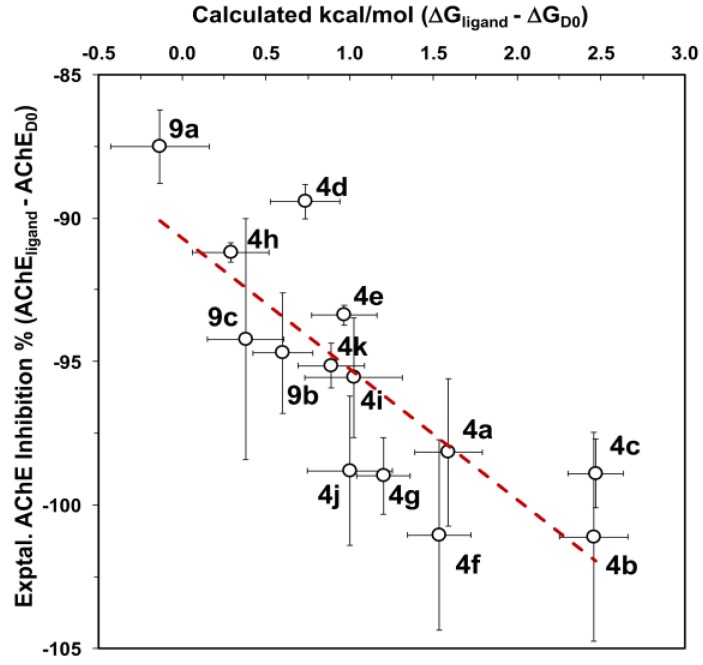
Free Energy vs. % AChE binding inhibition differences.

In terms of cholinergic activity, most analogs poorly inhibited (<10%) AChE and BuChE. The exception was isonipecotates **9a** and **9c**, which modestly inhibited (10–20%) both enzymes. As expected, the reference compounds (**Ga** and **Do**) differentially inhibited AChE (almost 60% for **Ga**, and 98% for **Do**) and BuChE (by about 30% by **Ga**, and 60% for **Do**). Despite the low enzyme inhibitory activities, we were encouraged by a finding that a linear correlation existed when calculated binding free energy (kcal/mol) differences and experimentally derived % AChE binding inhibition differences were plotted, as illustrated in Figure 6. This correlation was meaningful because it confirmed that our predictive computational model for AChE binding was on the right path and implied that the designed compounds yielded useful leads whose cholinergic shortfall could be improved by SAR (structure activity studies). We now know that π-π stacking may not be the only essential SAR element for AChE/BuChE inhibition. Rather, a combination of π-π interactions plus H-bonding or polar groups may prove useful.

## 4. Conclusions

Taken together, this preliminary report indicates that we have generated six (**4a**, **4d**, **4e**, **4f**, **4g**, **9b**) good leads with strong antioxidant and minimal AChE inhibition activities. SAR studies and additional pharmacological evaluations will be undertaken to determine if these molecules meet our ultimate poly-active molecules design goal. Note that the essence of our approach to drug design is to develop molecules that can modulate synergistic disease pharmacologies—ROS reductions are a good starting point. Regarding any additional experimental details/data (NMR, Mass, etc.), this manuscript is simply a short communication or preliminary report on compounds whose synthesis is already well established and appropriately documented in the included references.
